# Scientific Items

**Published:** 1886-05-20

**Authors:** 


### SCIENTIFIC ITEMS.

**Effect of 280,000 Pounds of Explosives 190 Miles Away.**—A Boston special to the *Tribune* says: The jocose assertion of the first director of Harvard College Observatory, Professor William Bond, that the ponderous foundation stone on which the great refractor is poised could not be moved even by an earthquake, has at last been disproved by observations taken there on the occasion of the recent explosion at Hell Gate. The method used by Prof. W. A. Rogers to develop the distance of vibration was the placing of a saucer of mercury on the solid cellar floor. In this mercury was a speck or flaw. Upon this point was brought to bear a microscope of 750 magnifying power, the spider line being in exact coincidence with the flaw. The first vibration perceived was about a thousandth of an inch, and recurred at intervals for nearly two minutes, the greatest swaying of the mercury being over a space of one five-hundredth of an inch.

The air line distance between the Observatory and Hell Gate is nearly 190 miles. Accurate time was kept at both points. This explosion of 140 tons of the most powerful explosives, with the estimated lifting power of two and a quarter trillion pounds, is one of the greatest triumphs of science the world has ever known.—*Med. and Surg. Reporter.*

**Experimental Yellow Fever.**—Dr. Carlos Finlay, of Havana, has published the results of several experiments he has made on the inoculability of yellow fever. He performed the operation, or rather got it performed for him by mosquitoes, which he caused first to sting a patient suffering from yellow fever and shortly afterward a healthy person who was to be (with his own consent) the subject of the experiment. He found that the disease was only inoculable from the third to the sixth day. When two mosquitoes were employed, so that a double dose was given, the symptoms of the experimental disease were somewhat more severe than when only a single mosquito was used. Of eleven cases of inoculation, six were efficacious, one doubtful, and four negative. The period of incubation varied from five to fourteen days; the symptoms consisted of headache, pyrexia, injection, with sometimes an icteric tint of the conjunctiva, and in some cases albuminuria. The fever lasted, as in the ordinary form, from five to twenty-one days. The author believes that this method of producing artificial yellow fever will ultimately be found very valuable as a prophylactic against the natural and dangerous form of the disease.—*Lancet.*

**How Far Light Penetrates Deep Sea Depths.**—G. C. Hodges, in *Scientific American*, says: This subject, referred to by one of your correspondents in your issue of March 20, has been carefully investigated by Messrs. Fol and Sarosin, of the Society of Physics and Natural History of Geneva, Switzerland. Without giving all the details, it was found that light penetrated fresh at depths of 170 meters (558 feet), and at that depth "the light, at mid-day, was about as strong as that of a clear moonless night." Similar experiments carried on in the Mediterrean led to the following conclusions: "In the month of March, in the middle of the day and in bright sunlight, the last glimmer of light comes at 400 meters (1,300 feet) below the surface." A full report of these investigations appeared in the *Photographic Times* of July 10 and October 9, 1885.

**Guthrie's Telephone.**—A contributor to the telephone controversy comes from Leesburg, Ohio, where it is reported that Mr. J. T. Guthrie experimented on the transmission of speech by electricity long before Bell received his now famous telephone patent of 1876. It is stated that Mr. Guthrie has now perfected a new form of telephone quite different from any previous device. A patent has recently been granted to him for a telephone which is operated by a direct instead of an induced current of electricity, as in other telephones. This instrument is not affected by the weather. The intensity of the current is regulated by a turn of the key. It is cheap, and applicable to any telegraph wire. The ticking of a watch is distinct over a three mile circuit, and speech is stated to be possible over a distance of a thousand miles. It is shortly to be given an extended test.—*Scientific American.*

**A Curious** application of the magnet is described in a French journal, the subject of it being a clock recently patented in France. In appearance the clock consists of a tambourine, on the parchment head of which is painted a circle of flowers corresponding to the hour-signs of ordinary dials. On examination two bees, one large and the other small, are discovered crawling among the flowers. The small bee runs rapidly from one to the other, completing the circle in an hour; while the large one takes twelve hours to finish the circuit. The parchment membrane is unbroken, and the bees are simply laid upon it: but two magnets, connected with the clock-work inside the tambourine, move just under the membrane; and the insects, which are of iron, follow them.—*Pop. Science News.*

**Walnut Hair-Dye.**—The juice of the walnut rind has been used from time immemorial as a hair-dye. Bernschen and Semper have recently communicated to the Berlin Chemical Society a method of preserving it for use in the shape of a hydro-glucoside, prepared as follows: The rinds of the ripe nut are digested in sulphuric ether until their coloring matter is extracted. A solution of chromic acid in water is added to the ether solution, and the mixture thoroughly agitated. The ether is then distilled off, and the residue purified by solution, first in hot ether, and afterward in a mixture of chloroform and petroleum ether, from which latter it is obtained in a crystalline form as hydrogen glucoside. This substance colors the hair and skin exactly as does the juice of the fresh rind.—*Ibid.*

